# Socioeconomic disparities in access to care and healthcare expenditures among U.S. adults: evidence from the Medical Expenditure Panel Survey, 2018–2022

**DOI:** 10.1097/MS9.0000000000005692

**Published:** 2026-09-17

**Authors:** Kolade O. Olatunde

**Affiliations:** Department of Public Policy (Health Policy Concentration), University of Arkansas, Fayetteville, AR, USA

**Keywords:** educational attainment, healthcare access, healthcare expenditures, health insurance coverage, Medical Expenditure Panel Survey (MEPS), poverty level

## Abstract

**Background:**

Socioeconomic disparities in health insurance coverage, income, and education are well-established determinants of healthcare access and spending in the United States. Less is known about how these disparities manifested during 2018–2022, a period marked by major disruptions to healthcare delivery during the COVID-19 pandemic. This study examined associations of insurance coverage and poverty level with access to care and of educational attainment with healthcare expenditures among U.S. adults.

**Materials and methods:**

Medical Expenditure Panel Survey (MEPS) Full-Year Consolidated files from 2018 to 2022 were pooled and analyzed. Survey-weighted logistic regression models were estimated for access-to-care outcomes, adjusting for age, sex, race/ethnicity, and calendar year, while accounting for MEPS’s complex survey design. Given the skewed distribution of expenditures, two-part models were used, combining logistic regression for any spending with gamma generalized linear models for expenditures among spenders.

**Results:**

Among adults under age 65, uninsured individuals had higher odds of delayed medical care and substantially lower odds of having a usual source of care compared with privately insured adults. Lower poverty levels were associated with progressively worse access outcomes. Educational attainment was strongly related to healthcare spending: adults with lower education were less likely to incur any expenditures, but among spenders, total costs were modestly higher and out-of-pocket spending consistently lower than among adults with a bachelor’s degree or higher.

**Conclusion:**

Socioeconomic disparities in healthcare access and expenditures persisted during 2018–2022, underscoring the need for policies that expand coverage, strengthen primary care access, and reduce cost-related barriers for disadvantaged populations.

## Introduction

Access to healthcare and its affordability remain significant and ongoing issues in the United States, especially as rising costs intersect with long-standing socioeconomic disparities^[^1,2^]^. Although much research has explored links between health insurance, poverty, education, and healthcare access, substantial gaps remain in understanding how these factors interact to affect access and financial strain during major systemic disruptions^[^3–5^]^. Less attention has been paid to how these relationships have evolved in recent years, especially amid unprecedented policy changes and shocks such as the COVID-19 pandemic, which has affected insurance coverage, healthcare delivery, and utilization.

HIGHLIGHTS

- Insurance coverage and poverty level were strongly linked to delayed medical care and not having a regular source of care among U.S. adults.
- Uninsured adults had significantly higher odds of delayed care and a much lower likelihood of having a regular source of care compared with privately insured adults.
- Income-related disparities in access to care persisted throughout the 2018–2022 period, including during the COVID-19 pandemic.
- Educational attainment was associated with both the likelihood of incurring healthcare costs and the amount of spending among those with positive expenditures.
- Adults with lower educational attainment were less likely to incur any healthcare spending but faced lower out-of-pocket costs when they sought care.

Healthcare access is usually defined as the prompt use of appropriate health services to achieve optimal health outcomes, encompassing aspects such as availability, accessibility, accommodation, affordability, and acceptability^[^6,7^]^. In studies, access is often gauged using indicators such as delays in medical care and a usual source of care, both of which are closely linked to unmet health needs, preventable hospital stays, and worse health outcomes[8]. Despite policy efforts to broaden coverage and improve access, these barriers still exist and tend to unfairly affect socioeconomically disadvantaged groups^[^9–11^]^.

Healthcare expenditures are another important aspect of equity, covering both overall payer spending and out-of-pocket costs paid directly by individuals. The United States still has the highest per-capita healthcare spending worldwide, but this does not ensure equitable access or financial protection[12]. Out-of-pocket costs remain a significant burden for many households and are closely associated with delayed care, skipped services, and financial hardship[13]. Understanding how socioeconomic status affects both access to care and healthcare spending is, therefore, essential for developing policies that address both cost control and equity.

Previous research consistently shows that uninsured adults are more likely to delay care and lack a usual source of care^[^14,15^]^, while people living in poverty face additional financial and structural barriers, even when insured[16]. Educational background also influences healthcare experiences by affecting health literacy, the ability to navigate the healthcare system, and patterns of service use[17]. However, much of the existing research examines these factors separately or focuses on single-year snapshots, which limits understanding of how their combined effects may change over time or with shifting policies and social conditions.

The period from 2018 to 2022 is a crucial window for studying these dynamics. This time frame includes pre-pandemic conditions, the peak of the COVID-19 pandemic, and the early recovery phase, during which healthcare delivery quickly shifted to telehealth, insurance coverage patterns changed, and many people faced economic instability. These contextual factors may have impacted traditional links between insurance status, poverty, education, and healthcare access, along with their effects on healthcare spending. However, few nationally representative studies explicitly examine this period to see whether and how socioeconomic disparities in access and spending persisted or evolved.

Specifically, this study addresses the following research questions:

1. How are health insurance coverage and poverty level associated with delayed medical care and a usual source of care among U.S. adults during 2018–2022?
2. How is educational attainment associated with total and out-of-pocket health care expenditures among U.S. adults over the same period?

By analyzing these relationships simultaneously and within a well-defined, impactful period, this study enhances the literature by explaining how established socioeconomic factors influencing healthcare access and expenditures function under systemic stress. This study adheres to the TITAN Guidelines (2025) for transparency and integrity in the use of artificial intelligence in research and reporting[18].

## Methods

### Study design and data source

This study used a cross-sectional, observational design with secondary data from the Medical Expenditure Panel Survey (MEPS) Household Component for the survey years 2018 through 2022. MEPS is conducted annually by the Agency for Healthcare Research and Quality (AHRQ) and aims to produce nationally representative estimates of the U.S. civilian, noninstitutionalized population. Pooling was conducted in accordance with AHRQ’s methodological guidance for multi-year MEPS analyses. The data files used in this study are publicly available and can be accessed through the AHRQ MEPS data portal: https://meps.ahrq.gov/mepsweb/data_stats/download_data_files.jsp.

### Study population

The analytic sample consisted of adults aged 18 years or older drawn from the MEPS. Respondents were included if they had valid data on the primary independent variables, including health insurance coverage, poverty level, and educational attainment, as well as the outcome measures of delayed medical care, having a usual source of care, and health care expenditures. Key sociodemographic covariates were also required. Observations with missing, inapplicable, or refused responses were excluded in an outcome-specific manner; consequently, analytic sample sizes varied slightly across models depending on data availability.

### Survey design and weighting

All analyses accounted for the complex multistage survey design of MEPS to ensure nationally representative estimates and valid statistical inference. Person-level sampling weights, strata, and primary sampling units (PSUs) were incorporated into all descriptive, bivariate, and multivariable analyses. When pooling data across the 2018–2022 survey years, person-level weights were divided by five in accordance with AHRQ recommendations to avoid overestimation of population totals.

### Measures

Health insurance coverage was measured using the MEPS insurance variable (INSURC), indicating coverage status over the calendar year and categorized as private insurance, public insurance, uninsured, or other coverage. Poverty level was assessed using the MEPS poverty category variable (POVCAT), which classifies family income relative to the federal poverty line. Educational attainment was measured using years of education completed (EDUCYR) and was treated as a continuous variable. Delayed medical care was assessed using the DLAYCA variable and was coded as a binary indicator of whether respondents delayed needed care during the year. Having a usual source of care was measured using the HAVEUS variable. Healthcare expenditures included total annual expenditures from all sources (TOTEXP) and out-of-pocket expenditures paid by individuals or families (TOTSLF), both measured in U.S. dollars.

### Statistical analysis

Survey-weighted means and proportions were used to describe sample characteristics, and survey-weighted cross-tabulations examined bivariate associations. Multivariable survey-weighted logistic regression models estimated associations between insurance coverage, poverty level, and access-to-care outcomes, with results reported as adjusted odds ratios and 95% confidence intervals. Due to substantial right skewness in expenditure data, generalized linear models with a gamma distribution and log link were used to examine associations between educational attainment and healthcare expenditures. Model diagnostics assessed multicollinearity and fit, and sensitivity analyses evaluated robustness across alternative specifications. Statistical significance was defined using a two-sided α of 0.05.

## Results

Across pooled MEPS Full-Year Consolidated files from 2018–2022, the analytic sample comprised 96 444 U.S. adults aged 18 years and older with positive person-level weights, representing an average annual U.S. adult population of approximately 230.9 million. The weighted mean age was 48.2 years, and 51.5% of respondents were female. The weighted racial and ethnic composition was 62.0% non-Hispanic White, 16.7% Hispanic, 12.0% non-Hispanic Black, 6.2% non-Hispanic Asian, and 3.0% non-Hispanic other or multiple races. Missing data were minimal for key outcomes, including delayed medical care (1.3%) and having a usual source of care (3.6%), as well as for educational attainment (0.7%). Overall, 7.9% of adults reported delayed medical care, while 72.0% reported having a usual source of care. The prevalence of delayed care declined steadily from 9.44% in 2018 and 9.42% in 2019 to 6.03% in 2022, whereas the prevalence of having a usual source of care remained relatively stable, with modest year-to-year variation.

### Insurance coverage, poverty level, and access to care

Survey-weighted logistic regression models were estimated for delayed medical care and having a usual source of care, adjusting for age, sex, race/ethnicity, and calendar year, with robust standard errors clustered at the MEPS PSU level. Results are summarized in Table 1 and presented separately for each access outcome in Tables 2 and 3.

**Table 1:** Adjusted associations with access-to-care outcomes (reference groups: private insurance; high income ≥400% FPL).

Predictor	Delayed care aOR (95% CI)	Usual source of care aOR (95% CI)
Public only vs private	0.95 (0.84–1.07)	1.21 (1.13–1.30)
Uninsured vs private	2.95 (2.67–3.26)	0.27 (0.25–0.30)
Poor vs high income	2.16 (1.93–2.41)	0.65 (0.61–0.70)
Near poor vs high income	2.06 (1.78–2.39)	0.82 (0.67–0.99)
Low income vs high income	2.22 (1.99–2.49)	0.78 (0.71–0.86)
Middle income vs high income	1.91 (1.78–2.05)	0.79 (0.74–0.85)

FPL, Federal Poverty Level.

**Table 2:** Adjusted associations between insurance coverage, poverty level, and delayed medical care.

Predictor	aOR	95% CI	*P*-value
Insurance coverage			
Private (ref)	1.00	—	—
Public only	0.95	0.84–1.07	0.39
Uninsured	2.95	2.67–3.26	<0.001
Poverty level			
High income (≥400% FPL) (ref)	1.00	—	—
Middle income (200–399% FPL)	1.91	1.78–2.05	<0.001
Low income (125–199% FPL)	2.22	1.99–2.49	<0.001
Near poor (100–124% FPL)	2.06	1.78–2.39	<0.001
Poor (<100% FPL)	2.16	1.93–2.41	<0.001

FPL, Federal Poverty Level.

Model notes. *N* (unweighted) = 72 381. Outcome: Delayed medical care (yes/no). Survey-weighted logistic regression adjusted for age, sex, race/ethnicity, and calendar year. Person-level weights applied; robust standard errors clustered by MEPS primary sampling unit (VARPSU).

**Table 3:** Adjusted associations between insurance coverage, poverty level, and having a usual source of care.

Predictor	aOR	95% CI	*P*-value
Insurance coverage			
Private (ref)	1.00	—	—
Public only	1.21	1.13–1.30	<0.001
Uninsured	0.27	0.25–0.30	<0.001
Poverty level			
High income (≥400% FPL) (ref)	1.00	—	—
Middle income (200–399% FPL)	0.79	0.74–0.85	<0.001
Low income (125–199% FPL)	0.78	0.71–0.86	<0.001
Near poor (100–124% FPL)	0.82	0.67–0.99	0.04
Poor (<100% FPL)	0.65	0.61–0.70	<0.001

FPL, Federal Poverty Level.

Model notes. *N* (unweighted) = 69 902. Outcome: Has a usual source of care (yes/no). Survey-weighted logistic regression adjusted for age, sex, race/ethnicity, and calendar year. Robust standard errors clustered by MEPS PSU. aOR = adjusted odds ratio.

### Delayed medical care

After adjustment, insurance coverage and poverty level were strongly linked to delayed medical care. Uninsured adults had nearly three times the odds of delaying care compared to those with private insurance (aOR = 2.95; 95% CI: 2.67–3.26; *P* < 0.001), while adults with public-only coverage did not differ significantly from those with private insurance (aOR = 0.95; 95% CI: 0.84–1.07). A clear income gradient was evident. Compared to high-income adults (≥400% Federal Poverty Level [FPL]), the odds of delayed care were significantly higher for adults who were poor (<100% FPL), near poor (100–124% FPL), low income (125–199% FPL), and middle income (200–399% FPL), with adjusted odds ratios between 1.91 and 2.22 (all *P* < 0.001). Adjusted predicted probabilities reflected these findings. The estimated probability of delayed care was 7.9% for privately insured adults, 7.5% for those with public-only coverage, and 19.7% for uninsured adults (Fig. 1). By income, predicted delayed care was 12.0% among poor adults compared to 6.0% among high-income adults.

**Figure 1.**
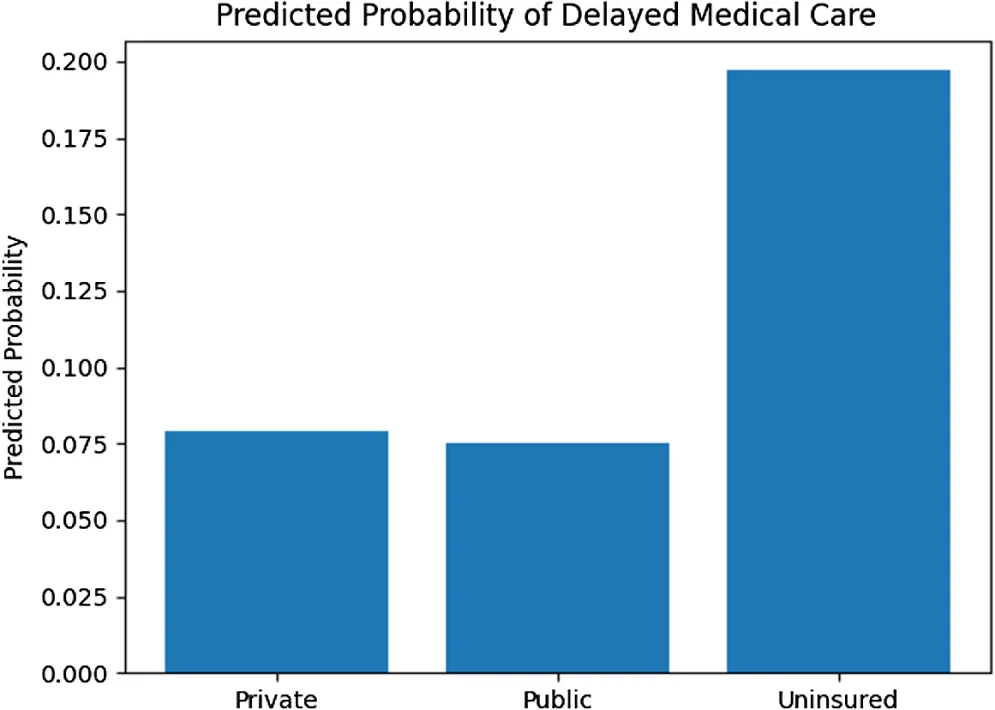
Predicted probability of delayed medical care by insurance status.

Predicted probabilities were derived from survey-weighted logistic regression models adjusting for age, sex, race/ethnicity, and calendar year, with robust standard errors clustered at the primary sampling unit level. Estimates represent average marginal predictions.

### Usual source of care

Insurance coverage was also strongly linked to having a usual source of care. Compared with privately insured adults, uninsured adults were much less likely to report a usual source of care (aOR = 0.27; 95% CI: 0.25–0.30; *P* < 0.001), while adults with public-only coverage had slightly higher odds (aOR = 1.21; 95% CI: 1.13–1.30; *P* < 0.001). Poverty status was also linked to a lower likelihood of having a usual source of care. Compared with high-income adults, those who were poor, near poor, low income, or middle income had significantly lower odds, with adjusted odds ratios between 0.65 and 0.82. Adjusted predicted probabilities showed that having a usual source of care was 69.1% among privately insured adults, 72.9% among those with public-only coverage, and 39.8% among the uninsured (Fig. 2). Across income levels, predicted probabilities ranged from 61.3% among poor adults to 69.9% among high-income adults.

**Figure 2.**
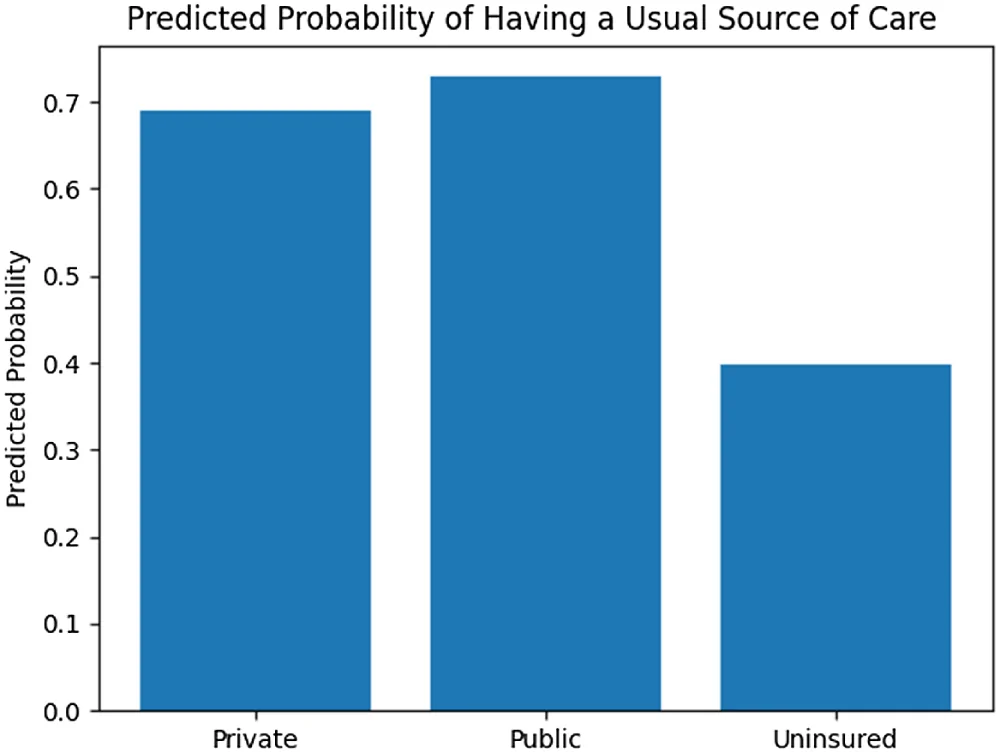
Predicted probability of having a usual source of care by insurance status, MEPS 2018–2022.

Predicted probabilities were estimated from survey-weighted logistic regression models adjusted for age, sex, race/ethnicity, and year.

### COVID-era context and temporal heterogeneity

To evaluate whether socioeconomic disparities changed during the COVID-19 period, interaction models compared 2018–2019 to 2020–2022, controlling for year indicators. Joint tests showed that poverty-related disparities differed significantly across periods for both delayed care (*P* < 0.001) and usual source of care (*P* = 0.003). Evidence for insurance-related differences was weaker: the insurance-by-period interaction was borderline-significant for delayed care (*P* = 0.049) and not statistically significant for usual source of care (*P* = 0.36). These results support treating the pooled period as contextually heterogeneous and highlight the importance of considering COVID-era access dynamics.

### Educational attainment and healthcare expenditures

Because healthcare expenditures were highly right-skewed, with a significant proportion at zero, two-part models were used for total expenditures (TOTEXP) and out-of-pocket expenditures (TOTSLF). The first part estimated the probability of any spending, while the second part modeled expenditure levels among spenders using a gamma distribution with a log link. Models were adjusted for age, sex, race/ethnicity, and year. Results are presented in Tables 4 and 5.

**Table 4:** Two-part model results for total healthcare expenditures (TOTEXP) by educational attainment, MEPS 2018–2022.

Part 1: Any total expenditures (>0)			
Education	aOR	95% CI	*P*-value
Bachelor’s degree or higher (ref)	1.00	—	—
Some college	0.70	0.64–0.76	<0.001
High school/GED	0.48	0.45–0.52	<0.001
Less than high school	0.51	0.48–0.54	<0.001
**Part 2: Total expenditures among spenders (Gamma, log link)**			
**Education**	**Exp(β)**	**95% CI**	***P*-value**
Bachelor’s degree or higher (ref)	1.00	—	—
Some college	1.06	1.01–1.11	0.015
High school/GED	1.03	0.98–1.08	0.26
Less than high school	1.18	1.03–1.35	0.014

Model notes. *N* (Part 1) = 96 444. *N* (Part 2) = 82 917. Two-part survey-weighted model. Part 1 estimated via logistic regression; Part 2 estimated using Gamma generalized linear model with log link. Models adjusted for age, sex, race/ethnicity, and year; SEs clustered by PSU. Exp(β) represents percent differences in mean expenditures among spenders.

**Table 5:** Two-part model results for out-of-pocket expenditures (TOTSLF) by educational attainment, MEPS 2018–2022.

Part 1: Any out-of-pocket expenditures (>0)			
Education	aOR	95% CI	*P*-value
Bachelor’s degree or higher (ref)	1.00	—	—
Some college	0.71	0.67–0.75	<0.001
High school/GED	0.51	0.48–0.53	<0.001
Less than high school	0.49	0.46–0.52	<0.001
**Part 2: Out-of-pocket expenditures among spenders (Gamma, log link)**			
**Education**	**Exp(β)**	**95% CI**	***P*-value**
Bachelor’s degree or higher (ref)	1.00	—	—
Some college	0.82	0.76–0.87	<0.001
High school/GED	0.72	0.64–0.82	<0.001
Less than high school	0.65	0.60–0.71	<0.001

Model notes. *N* (Part 1) = 96 444. *N* (Part 2) = 71 206. Two-part survey-weighted model with adjustment for age, sex, race/ethnicity, and year. Estimates are descriptive and reflect associations rather than causal effects.

Probability of any expenditure and expenditure levels among spenders.

Compared with adults holding a bachelor’s degree or higher, those with lower levels of educational attainment were significantly less likely to incur any healthcare expenditures, including both total and out-of-pocket costs. Adults with less than a high school education had approximately 50% lower odds of reporting any total or out-of-pocket expenditures (*P* < 0.001 for all comparisons). Among adults with positive expenditures, spending patterns differed by expenditure type. For total healthcare expenditures, adults with less than a high school education spent 18% more on average than those with a bachelor’s degree or higher, while adults with some college education spent 6% more. In contrast, out-of-pocket expenditures were substantially lower among adults with lower educational attainment, with reductions ranging from 18 to 35% relative to the highest education group. When both parts of the models were combined, adjusted predicted mean total expenditures ranged from $7292 among adults with a high school education or GED to $8344 among those with less than a high school education. Predicted mean out-of-pocket expenditures increased monotonically with education, from $721 among adults with less than a high school education to $1234 among those with a bachelor’s degree or higher (Fig. 3).

**Figure 3.**
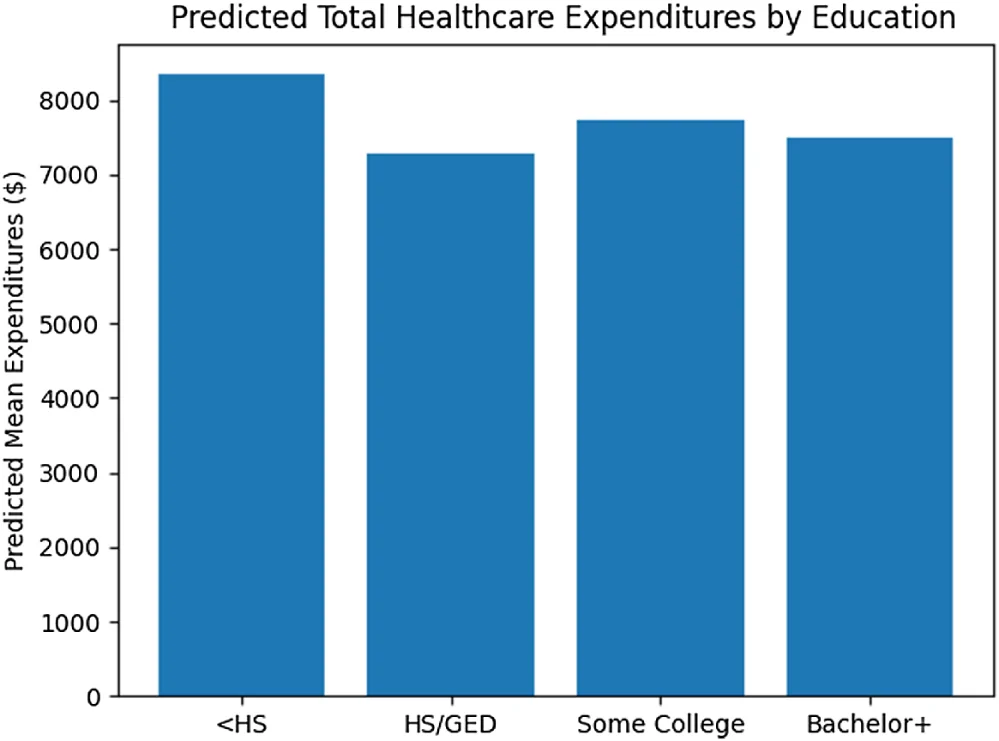
Predicted mean annual total healthcare expenditures by educational attainment, MEPS 2018–2022.

Predicted means were calculated using a two-part model consisting of (1) a survey-weighted logistic regression for any expenditures and (2) a Gamma generalized linear model with a log link among spenders, adjusted for age, sex, race/ethnicity, and year.

## Discussion

Using pooled, nationally representative MEPS data from 2018 to 2022 and models that account for MEPS’s complex design, we identified significant socioeconomic disparities in access to care and clear gradients in healthcare expenditures. Insurance coverage and poverty level were strongly associated with both delayed medical care and having a usual source of care, even after adjusting for age, sex, race/ethnicity, and year. These patterns are consistent with previous research showing that insurance and limited economic resources are major barriers to timely care and stable entry points into the healthcare system. Notably, the significantly lower likelihood of having a usual source of care among uninsured adults aligns with evidence that insurance and stable primary care relationships collaboratively enhance access, preventive care, and continuity^[^19,20^]^.

Importantly, the 2018–2022 period includes significant disruptions in healthcare delivery and survey operations caused by the COVID-19 pandemic. Our year-specific patterns – such as lower reported delayed care in 2020–2022 compared with 2018–2019 and minor changes in usual source of care – should be viewed in this context. Early in the pandemic, widespread delays in or avoidance of care were documented nationally by CDC surveillance, and care-seeking behavior and service availability changed quickly[21] (e.g., cancellations, telehealth use, and public avoidance of in-person visits). Additionally, MEPS field operations and data collection shifted in 2020, including changes that AHRQ has advised analysts to consider for pooling and trend analyses[22]. These contextual factors may help explain why access indicators can show unexpected changes during the pandemic years (for example, reductions in reported delayed care may reflect deferred demand, perceived changes in need, altered service availability, or reporting differences).

Our analysis of expenditures used a two-part modeling approach to address the common skewness and the concentration of zeros in cost outcomes. Two-part models are widely recommended in health economics and applied outcomes research because they distinguish between (1) the likelihood of any spending and (2) the amount spent among those with positive expenditures – often employing a gamma log-link GLM in the second stage^[^23,24^]^. In this study, educational attainment was consistently correlated with the probability of any spending (lower-education groups were less likely to have any annual expenditures). However, among those who did spend, total expenditure was slightly higher for some lower-education groups, while out-of-pocket expenses tended to be lower. This pattern is logical and aligns with structural mechanisms: lower-education groups may face a higher burden of illness and rely more on publicly funded care, which shifts costs away from out-of-pocket payments, whereas higher-education groups are more likely to use discretionary services and incur greater cost-sharing. Evidence from broader policy and coverage research supports the idea that coverage expansions and public insurance can reduce out-of-pocket burden and the risk of catastrophic spending[25].

Recent scholarship highlights both the rapid growth of large language model (LLM) applications in medicine and persistent concerns regarding accuracy, bias, transparency, and ethical use[26]. Consistent with these recommendations, our study employed a generative chatbot in a clearly bounded methodological role, with all analytic decisions and interpretations independently reviewed by the research team, and no identifiable or sensitive data were entered into the model. The study’s outcomes are therefore grounded in validated data and established analytic procedures, with LLM assistance serving as a complementary tool rather than a driver of results.

### Policy implications and conclusion

The findings of this study highlight several important policy and practice priorities for reducing persistent socioeconomic disparities in healthcare access and affordability. First, closing gaps in health insurance coverage, particularly among non-elderly adults, remains critical, given the strong association between uninsurance and both delayed medical care and the absence of a usual source of care. Expanding and stabilizing coverage options may help mitigate access barriers and promote continuity of care. Second, strengthening access to primary care homes and safety-net providers may be especially beneficial for low-income and uninsured populations, as these settings can serve as stable points of entry into the healthcare system and buffer against access disruptions during periods of system shock, such as public health emergencies[27]. Third, observed differences in out-of-pocket expenditures by educational attainment suggest that affordability protections, including cost-sharing subsidies, Medicaid continuity, and targeted assistance for underinsured populations, remain essential. Although lower-education groups incurred lower out-of-pocket spending on average, they may still face substantial financial strain due to limited resources and competing demands.

In conclusion, an analysis of pooled MEPS data from 2018 to 2022 demonstrates that insurance coverage and poverty level are strongly associated with delayed medical care and with having a usual source of care among U.S. adults, even after accounting for sociodemographic factors and the complex survey design. Educational attainment was also linked to healthcare expenditures, underscoring enduring socioeconomic inequities in access and affordability during a period of major healthcare system disruption.

## Data Availability

The datasets analyzed during the current study are publicly available from the Medical Expenditure Panel Survey (MEPS) database provided by the Agency for Healthcare Research and Quality (AHRQ). The data can be accessed at: https://meps.ahrq.gov/mepsweb/data_stats/download_data_files.jsp.

## Research registration unique identifying number (UIN)

Research registry number: Not applicable. This study involves secondary analysis of publicly available, de-identified data from the Medical Expenditure Panel Survey (MEPS) and therefore does not require prospective registration

## Assistance with the study

The author would like to thank the organizers of the 2025 Arkansas Summer Research Institute (ASRI) for providing a collaborative environment and training resources that supported the development of this project. The author also acknowledges the support of faculty mentors and colleagues who provided constructive feedback during the early phases of the study.
